# Lifestyle Interventions for Prevention and Management of Diet-Linked Non-Communicable Diseases among Adults in Arab Countries

**DOI:** 10.3390/healthcare11010045

**Published:** 2022-12-23

**Authors:** Maryam Naveed Muhammad Tariq, Lily Stojanovska, Ayesha S. Al Dhaheri, Leila Cheikh Ismail, Vasso Apostolopoulos, Habiba I. Ali

**Affiliations:** 1 Department of Nutrition & Health, College of Medicine and Health Sciences, United Arab Emirates University, Al Ain 15551, United Arab Emirates; 2 Institute for Health and Sport, Victoria University, Melbourne, VIC 3030, Australia; 3 Department of Clinical Nutrition and Dietetics, College of Health Sciences, University of Sharjah, Sharjah 27272, United Arab Emirates; 4 Nuffield Department of Women’s & Reproductive Health, University of Oxford, Oxford OX1 2JD, UK

**Keywords:** Arab adults, Arab countries, diabetes, cardiovascular diseases, lifestyle interventions, Middle East, NCDs, nutrition programs, obesity, overweight

## Abstract

The increased incidences of diet-related non-communicable diseases (NCDs) such as diabetes, obesity, and cardiovascular diseases among adults are becoming the chief public health concern in most Arab countries. Economic expansion has contributed to a nutrition shift from a traditional seasonal diet to Westernized eating habits coupled with a sedentary lifestyle. Despite the rising concern for NCD mortality, public health policies are inadequately addressed. This narrative review aims to discuss the effectiveness of nutritional interventions focusing on diet and physical activity in the management of NCDs among Arab adults. A comprehensive literature search was performed using different database platforms such as Cochrane reviews, Scopus, and PubMed for articles published between 1 December 2012 and 31 December 2021. Fifteen recent research articles addressing NCDs, mainly diabetes and obesity, from different Arab countries were included in this review. Structured lifestyle interventions involving behavioral therapy approaches and personalized goals for diet and physical activity were found to improve specific health outcomes in most studies. Significant improvements in health outcomes were reported for longer-duration interventions with follow-ups. A combination of both online and face-to-face sessions was found to be effective. It is important to identify barriers to physical activity for a culturally acceptable lifestyle intervention and conduct further studies to evaluate interventions for the long-term maintenance of health outcomes.

## 1. Introduction

Globally, non-communicable diseases (NCDs), such as cardiovascular diseases (CVD) and diabetes, are responsible for around 41 million deaths annually [1]. In 2016, unhealthy diets were categorized as the second risk factor contributing to the global burden of diseases, accounting for nearly 11 million casualties in 2017 [2,3]. Physical inactivity was also rated as one of the chief factors causing global mortality, amounting for over 1.3 million global deaths [4].

Regionally, increased incidences of type-2 diabetes, obesity, cancer, CVD, and other diet-related NCDs among adults are becoming the main public health concern in most Arab countries [5,6]. The intake of energy-dense foods is negatively influencing public health care and social and economic practices [5,6]. In 2008, 1.2 million deaths were recorded due to NCDs in Arab countries, amounting for 60% of all mortalities [7,8]. Furthermore, the mortality percentages from NCDs in fourteen Arab countries ranged from 73% to 89%, with an average of 80.4%, as per a 2022 World Health Organization (WHO) report [9]. In 86% of the Arab countries, the risk of premature death (aged between 30 and 70 years) due to NCDs averages around 21% [9]. Moreover, a high body mass index (BMI), increased blood pressure, physical inactivity, and an unhealthy diet are risk factors that are known to contribute to the highest attributable disability-adjusted life years (DALYs) for NCDs [10,11].

Populations in the Arab region have experienced a nutritional shift characterized by a transition from a traditional, more diverse, and seasonal diet high in fruits, whole grains, and vegetables to Westernized diets that are high in refined carbohydrates, trans fats, saturated fats, salt, and sugar [6,7]. High incomes, economic expansion, rapid urbanization, and the globalization of marketing and trade also contribute to this nutritional transition [5,12]. Moreover, the economic development in the Arab region following the oil discovery led to huge changes in lifestyle, eventually leading to diet-related chronic diseases [13].

Considering the gravity of the rising problem of nutrition-related NCDs, studies have addressed the requirements for a healthy lifestyle and culturally adaptable dietary patterns to address the public health concerns in Arab countries [6,14,15]. Furthermore, several Arab countries held regional, international, and national gatherings to discuss possible solutions [16]. These discussions recognized the need to establish interventions and public health policies, addressing the variable risk factors of non-communicable diseases, entailing nutrition, dietary habits, and behavior changes [16]. Implementing such interventions and guidelines would rely on current scientific evidence for their cost-effectiveness, efficacy, and feasibility [10].

Despite the rising concern and the risk factors for NCD mortality, public health policies are inadequately addressed in the Arab regions [17]. Moreover, the efficacy and feasibility of lifestyle-modification programs in the primary care setting for increased physical activity, healthy eating habits, and managing weight to curb diet-related NCDs in Arab countries are still being disputed by numerous healthcare policymakers and practitioners [18,19]. Lifestyle interventions are defined as having at least one additional component, such as behavioral therapy, counseling, or stress management, in addition to both nutritional and physical activity components [20]. Several studies reported sociocultural barriers to a healthy lifestyle among the Arab population, particularly among women [21,22,23,24,25]. Lifestyle intervention programs should be adapted to cultural and religious practices to be effective [26]. Hence, there is a need for community-based and culturally acceptable interventions in Arab countries [26,27,28]. To date, only a few review-based studies have been conducted in Arab countries, focusing on interventional programs, limited to physical activity only [25,29,30] or interventions led by specific healthcare providers such as pharmacists [31], and targeting different NCDs. However, such studies could not determine whether physical activity alone had a considerable impact on the reported health outcomes or if it had an impact when combined with other aspects of a multicomponent intervention, such as dietary changes [25,29,30,31]. Henceforth, this narrative review discusses the effectiveness of lifestyle interventions focusing on diet and physical activity in the prevention and management of NCDs, such as type-2 diabetes and obesity, among adults in Arab countries. It also provides insights into improving health outcomes and recommendations for enhancing prospective interventions in this region.

## 2. Materials and Methods

**Figure 1:**
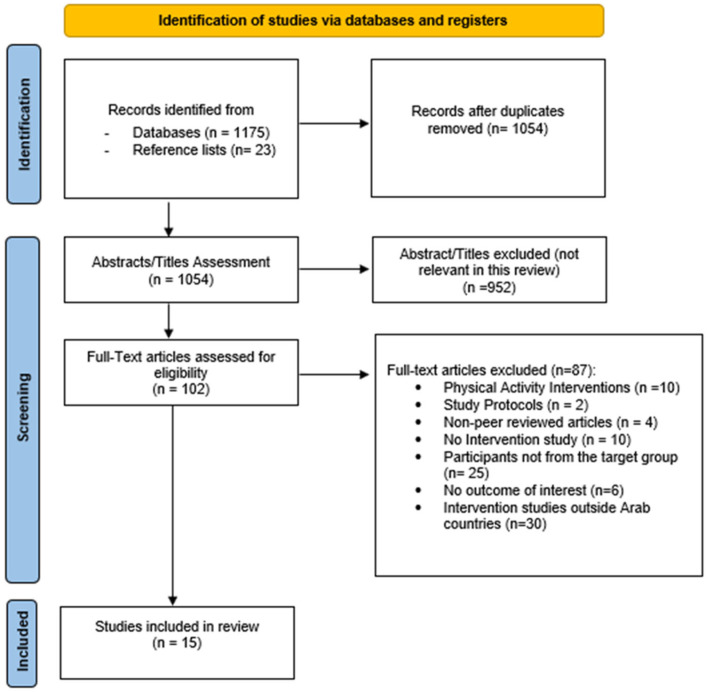
Flow chart of included and excluded studies.

The Arab League is comprised of 22 countries, namely Syria, Oman, the United Arab Emirates, Saudi Arabia, Mauritania, Bahrain, Lebanon, Yemen, Sudan, Iraq, Libya, Egypt, Jordan, Palestine, Djibouti, Qatar, Somalia, Morocco, Comoros, Tunisia, Algeria, and Kuwait [25]. A comprehensive literature search was performed using the Google Scholar, ResearchGate, SpringerLink, Cochrane reviews, Scopus, and PubMed database platforms for relevant articles. Articles that were published in the past 10 years from 1 December 2012 to 31 December 2021 were considered in the review. The search terms used in combination included ‘NCD prevention interventions’ OR ‘nutrition programs’ OR ‘nutrition interventions’ OR ‘lifestyle interventions’ OR ‘diabetes prevention programs’ OR ‘obesity prevention programs’ OR ‘heart disease programs’ OR ‘hypertension programs’ AND ‘Middle East’ OR ‘Arab countries’ OR ‘Arab Adults’. In addition, the names of some Arab countries (such as ‘Egypt’ OR ‘Jordan’ OR ‘Oman’ OR ‘United Arab Emirates’ OR ‘Bahrain’ OR ‘Qatar’ OR ‘Saudi Arabia OR ‘Tunisia’) were included in the search to identify possible missed articles. The articles obtained from the search were assessed for topic relevance and hand-reviewed to further find related publications. Only articles that involved interventions for adults (18 and above) to combat diet-related NCDs, such as type 2 diabetes, obesity, or heart diseases, were included in this study. Studies evaluating different parameters as health-related outcomes were included, such as physical activity and dietary habits (or energy intake). Randomized and non-randomized studies involving intervention groups (with diet and/or physical activity) and control groups (without intervention or with placebo) were also included. In total, 1198 articles were identified in the initial search. After the removal of 144 duplicates, 1054 titles or abstracts were screened, where 952 of them were not deemed to be relevant and were excluded. The remaining 102 full-text articles were further reviewed for eligibility, leading to the elimination of 87 full-text articles due to the inclusion criteria, as outlined in Figure 1. Therefore, 15 research articles were included in this narrative review.

## 3. Results

Fifteen lifestyle-intervention-based articles representing eight Arab countries, namely Bahrain [32], Egypt [33], Oman [34], the occupied Palestinian territories [35], Qatar [36], Saudi Arabia [24,28,37,38,39,40], Tunisia [41], and the United Arab Emirates (UAE) [21,26,42], were incorporated in this review paper. The summarized data from the incorporated studies, including the region where the study was performed, the study design, the target group, the sample size, and the intervention characteristics (the name, duration, and measured components or health outcomes) are shown in Table 1.

### 3.1. Characteristics of Lifestyle-Intervention-Based Studies

Seven studies focused on intervention programs to combat type 2 diabetes [24,33,34,35,36,37,42], two studies focused on overweight/obesity [26,38], three studies focused on both prediabetes/diabetes and obesity [21,28,39], two studies were workplace interventions on diet-related NCDs in general [32,41], and only one intervention focused on CVD [40]. Of these, four studies included only females as participants [21,24,26,40]. Hence, most identified NCD prevention interventions were related to diabetes and obesity.

The intervention-based studies had different study designs, including randomized controlled trials [28,34,36,38,40,42], non-randomized studies [26], quasi-experimental studies [37,41], cohort studies [32,33], an observational study [35], and other intervention studies [21,24,39]. Most studies addressed programs that were delivered face to face [21,24,28,32,33,35,36,38,39,40,41]. Some studies in the UAE, Saudi Arabia, and Oman reported both telephone or mobile-based and face-to-face interventions [34,37,42]. A UAE-based study by Ali et al. was completely mobile-based [26]. All studies included participants of either local or Arab ethnicity.

In general, the interventions provided participants with education on diet and physical activity [21,24,26,28,32,33,34,35,36,37,38,39,40,41,42]. The activities involved exercise sessions, diet counseling, and health education delivered by a team of dietitians, physiotherapists, health educators, and researchers [21,24,26,28,32,33,34,35,36,37,38,39,40,41,42]. The diabetes-based intervention studies used the American Dietetic Association food exchange system to calculate the carbohydrate intake from patients’ food records, including dairy, fruits, vegetables, starches, and other carbohydrates [36,39,42]. Similarly, to assess a person’s physical activity level, several studies were reliant on the WHO recommendation for adults aged 18 to 64 years to perform at least 150 min of moderate-intensity physical activity or at least 75 min of vigorous-intensity physical activity each week or a combination of both [33,34,39,41].

Some interventions were work-site initiatives [32,41], while others were community-based interventions [33,40], and most were conducted in healthcare settings [21,24,28,34,35,36,37,38,39,42]. Only one study by Ali et al. was performed among university students from 18 to 35 years [26]. The number of educational sessions given to the participants ranged from one [35,40] to four [36], eight [38,42], or forty-eight sessions [33]. The durations of these interventions were diverse, ranging from four hours [35] to three months [21,38,40], four months [26], six months [24,32,37,39,42], one year [28,33,34,36], or up to 3 years [41]. Only two studies in the UAE performed one-year post-intervention follow-ups [21,42].

**Figure 2:**
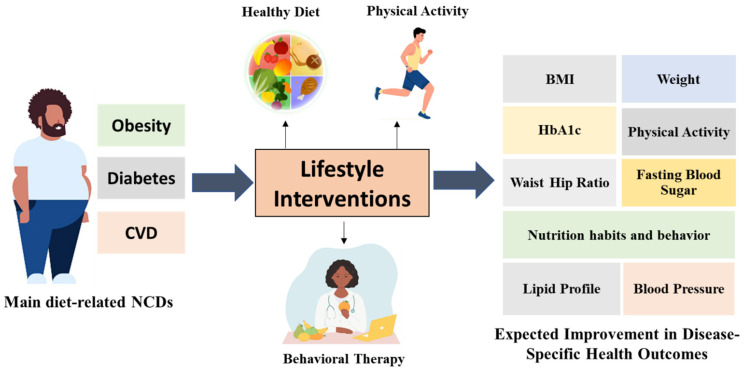
Lifestyle interventions for diet-linked NCD management and their impacts on health outcomes.

Common health markers were evaluated in nearly all studies, with the inclusion of BMI (body mass index) in all studies [21,24,26,28,32,33,34,35,36,37,38,39,40,41,42]. Specific health outcomes were assessed according to the disease in question. For instance, Khouja et al. [40] measured the Framingham risk score (FRS), in addition to other parameters, to assess the 10-year risk of heart disease, categorized into high (≥20% FRS), moderate (10%–19% FRS), and low risk (<10% FRS). Figure 2 shows a schematic diagram of the expected impacts of lifestyle interventions on selected health outcomes.

### 3.2. Assessment of Diet and Physical Activity Levels

Nutrition knowledge and behaviors were mainly evaluated through standardized questionnaires corresponding to each program [21,24,26,33,35,36,37,41]. The questionnaires were translated into Arabic to suit programs’ needs [24,26,36,37]. A study by Al-Hamdan and his colleagues [24] assessed dietary intake using the Food Frequency Questionnaire (FFQ) [24]. The accuracy of the questionnaire was tested using approaches such as criterion-related validity, test–retest reliability, and internal validity [24]. On the other hand, Alfawaz et al. used 24 h dietary recall to assess overall calorie, micronutrient, and macronutrient consumption by applying a validated computerized food database, i.e., “ESHA—the Food Processor Nutrition Analysis program” [39]. A study in the UAE by Sadiya et al. involved both dietary recall and FFQ [21]. Another study by Bhiri and colleagues in Tunisia assessed dietary behavior using the default daily intake of five servings of vegetables and fruits [41].

Physical activity was also mainly evaluated through standardized questionnaires [24,26,33,34,39,41]. One such study in Oman conducted by Alghafri et al. involved the use of the Global Physical Activity Questionnaire (GPAQ) to evaluate self-perceived PA changes [34]. Moreover, this study used pedometers and accelerometers to objectively assess PA after one year of intervention [34].

### 3.3. Impacts of Lifestyle-Based Interventions on Dietary Habits and Physical Activity Levels

All studies included an educational aspect related to diet and physical activity. Twelve studies assessed nutrition knowledge, behaviors, and physical activity levels [21,24,26,32,33,34,35,36,37,39,41,42]. Improved health outcomes were reported in most interventional studies [21,26,35,36,37,41].

The interventions in the studies conducted in Palestine and Saudi Arabia by Rashed et al. and Sani et al. were associated with significant improvements in diabetes knowledge among participants (*p* ≤ 0.001) [35,37]. Similarly, studies in Qatar, Tunisia, and the UAE by Mohamed et al., Bhiri et al., Sadiya et al., and Ali et al. reported significant improvement in dietary habits, behavior, and knowledge (*p* < 0.05) [21,26,36,41]. A study by Metwally et al. showed significant improvements in the mean scores of the studied behaviors compared to their pre-education levels, including dietary habits and physical activity [33]. Similarly, there were reductions in barriers related to diet, physical activity, medication adherence, and blood glucose monitoring in Egypt (*p* < 0.001) [33].

Other studies assessed the intake of specific food groups and nutrients [24,32,39,41,42]. A study in Saudi Arabia by Alfawaz and colleagues reported significant improvements in recommended dietary intake in the intervention group compared to the control group, especially in total carbohydrates (*p* = 0.003); dietary fiber (*p* = 0.002); and some micronutrients, such as vitamins B2 (*p* = 0.01), B3 (*p* < 0.001), B12 (*p* = 0.041), B6 (*p* < 0.001), vitamin E (*p* = 0.003), phosphorus (*p* < 0.001), copper (*p* = 0.03), potassium (*p* = 0.01), magnesium (*p* < 0.001), sodium (*p* = 0.01), and iron (*p* = 0.01) [39]. Worksite-intervention-based studies in Tunisia [32] and Bahrain [41] reported increments in fruit and vegetable intake among their respective participants after three years and six months of the programs [32,41]. A healthcare-setting-based intervention study in the UAE by Abdi et al. also reported increased fruit intake in the intervention but not in the control group after six months [42]. However, vegetable intake did not significantly improve in either group six months after the intervention started [42]. Healthcare-setting-based studies in the UAE and Saudi Arabia by Abdi et al. and Al-Hamdan et al. showed significant reductions in refined carbohydrate and total calorie intakes [24,42]. A study in Bahrain by Al Saweer et al. indicated a decreased intake of fat among employees, similar to the study in Saudi Arabia by Al-Hamdan et al., which also found significant reductions in fat intake in both the intervention and control groups after six months [24,32].

In the worksite- and healthcare-setting-based interventions conducted in Bahrain and Egypt by Al Saweer et al. [32] and Metwally et al. [33], significant increases in physical activity (*p* < 0.01) were reported among participants 6 and 12 months after the intervention [32,33]. A study by Bhiri et al. in Tunisia reported significant improvements in physical activity behaviors (*p* < 0.001) in both the intervention and control groups after the three-year worksite intervention [41]. Studies in Saudi Arabia and the UAE by Alfawaz et al. and Ali et al. showed that the intervention groups had significant improvements in moderate and vigorous physical activity levels and their frequency [26,39]. However, a study by Al-Hamdan et al. [24] in Saudi Arabia found no significant difference in physical activity levels in females when comparing within and between the groups [24]. Moreover, a UAE-based study by Abdi et al. [42] evaluated self-reported physical activity and found a non-significant increase in physical exercise levels (min/day) over the six month intervention period [42]. Further, as previously mentioned, a study by Alghafri et al. used pedometers and GPAQ to report changes after 12 months [34]. Although both groups showed constants increases in physical activity levels, the intervention group experienced a considerably greater mean increase from baseline than the control group at 12 months [34].

## 4. Discussion

This review evaluates the effectiveness of lifestyle interventions among adults in the Arab region based on the interventions’ effects on modifiable health indicators. Overall, intensive lifestyle interventions involving behavioral therapy approaches and personalized goals related to diet and physical activity were found to improve specific health outcomes in most studies [21,24,28,32,33,35,36,38,39,41,42].

The majority of NCD prevention programs addressed diabetes, prediabetes, overweight, and obesity [21,24,26,28,33,34,35,36,37,38,39,42]. The prevalence rates of diabetes and obesity in the Arab region, particularly in the Gulf Cooperation Council countries (the UAE, Bahrain, Qatar, Oman, Saudi Arabia, and Kuwait) are amongst the highest in the world [29,43,44]. According to the 2021 report by the International Diabetes Federation, the diabetes incidence rates in Arab countries varied from 6 to 25% [45]. Obesity prevalence rates, in contrast, have reached up to 40% and are regarded as a common risk factor in diabetes occurrence [46,47,48,49]. The main contributing factors are undoubtedly linked to a person’s lifestyle, including unhealthy dietary behaviors and a lack of physical activity [29,43,44,46,48,49]. Hence, interventions are aimed to focus on diabetes and obesity management to reduce the burden of such diseases on the health and economic systems in Arab countries over the coming years [29,43,44,48,49].

Intervention studies in Tunisia, Bahrain, Saudi Arabia, Egypt, and the UAE that involved and assessed both dietary habits and physical activity behaviors showed improved health outcomes [26,32,33,39,41]. Although some studies assessed dietary behavior and various habits, physical activity was not assessed as a health indicator [21,35,36]. In contrast, a study in Oman by Alghafri et al. focused on and assessed physical activity, but not dietary habits, and did not find significant changes in body weight, BMI, or HbA1c between the two groups after 12 months, despite being a multicomponent intervention that used a behavioral therapy approach [34]. Likewise, previous studies in obese patients have shown that interventions focusing only on physical activity may have small to modest impacts on body weight compared to a combination of both dietary and exercise-based interventions [50,51,52,53,54,55]. The latter studies showed that participants achieved around 5–11% weight loss with improvements in controlling obesity-linked comorbidities such as asthma, osteoarthritis, or metabolic anomalies linked with metabolic syndrome [50,51,52,53,54,55]. A systematic review evaluating 66 programs found that a combination of nutrition and physical activity programs was successful at reducing the incidence of diabetes and enhancing cardiometabolic risk factors in people at elevated risk [56]. Combining diet and exercise is most favorable for improving metabolic regulation and lowering body weight compared to diet or exercise alone [57,58,59,60]. This pairing regulates energy intake and creates a negative energy balance by increasing the expenditure of energy [57,58,59,60]. Hence, this emphasizes the need for multicomponent behavioral interventions to improve health outcomes.

Studies in the UAE and Saudi Arabia also reported significant weight loss (≥4–5%) among participants post-intervention, with program durations varying from 12 weeks to a year [21,28,38]. A one-year follow-up study by Sadiya et al. revealed sustained weight loss and improvement in other health outcomes, such as HbA1c, which was further reduced compared to the post-intervention results after three months [21]. Programs entailed strict diet plans (1200–1500 kcal/day), nutrition modification (total dietary fat < 30% of energy and fiber intake of 15 g/1000 kcal), physical activity (≥150 mins/week or ≥5000 steps/day), and behavioral therapy to achieve targeted weight loss of 5% more [21,28,38]. Moreover, these studies involved intensive lifestyle interventions that incorporated behavioral change by including individualized consultations as per participants’ needs when making customized goals and sessions educating patients on the self-monitoring and self-management of their respective diet-related NCDs [21,28,38]. Furthermore, lifestyle intervention studies in Egypt, Qatar, Saudi Arabia, and Tunisia [28,33,34,36,41] conducted for a year or more revealed significant improvements in weight, BMI [28,33,36], and/or significantly improved health outcomes (lipid profile, blood pressure, HbA1c, diet, physical activity, and nutrition knowledge) [33,36,41]. A review involving eight studies revealed that significant longer-term weight loss was observed after a year of combined behavioral weight management interventions involving diet and physical activity [53]. Hence, intensive lifestyle interventions inducing behavioral change for a year or more, coupled with regular follow-ups, could improve health outcomes, such as weight or BMI, as shown in previous studies [50,53,61,62,63,64,65,66].

Most programs were performed face-to-face [21,24,28,32,33,35,36,38,39,40,41]. Some studies entailed both telephone/mobile-based and face-to-face interventions [34,37,42], whereas only one study was completely mobile-based [26]. Behavioral therapy (nutrition education, a cognitive behavior approach, goal setting, and monitoring) is an essential component that was carried out by qualified dieticians and healthcare professionals either in-person or via technology (individual or group sessions) [59,60,67,68,69]. The use of technology (such as phone calls, phone apps, social media, online appointments, and online meeting sites) can be a useful alternative [59,60,67,68,69]. Hence, interventions with both online and face-to-face delivery modes showed improved behavioral outcomes [34,37,42]. This was similar to previous studies in the United States (US), Canada, the Netherlands, and Australia that evaluated the modes of delivery of interventions and reported that a combination of both online and face-to-face sessions is effective and convenient, considering the current COVID situation, where remote work is a valid option [70,71,72,73]. This is supported by a recent study evaluating the effectiveness of different delivery strategies of weight loss programs, where more participants in the hybrid app and face-to-face program lost 5% or more weight in comparison to the app group alone, demonstrating beneficial outcomes in supporting health experts while decreasing their workloads [74].

Most of the studies reported herein used questionnaires to assess physical activity levels or dietary habits and behavior, which can result in subjective responses by the participants [21,24,26,33,35,36,37,41]. Moreover, two randomized control trial based studies claimed to have assessed self-perceived or self-reported physical activity after the completion of the program [34,42]. Thus, bias related to self-reports is the main limitation of these studies. This highlights the need for standardized and validated measures for assessment to ensure consistent reporting and for comparison between studies [23,25]. Standardized interviews can be performed when managing the questionnaire to reduce under- or overestimating and guessing responses, which can occur when participants complete questions by themselves [75].

Studies in Palestine and Egypt reported sociodemographic differences between participants, where more women compared to men were found to be obese and showed limited participation in physical activity during program implementation due to sociocultural and environmental barriers [33,35]. Physical inactivity is becoming increasingly prevalent, particularly among Arab women, where sociocultural and economic barriers were reported [21,22,23,24,25]. In developing Arab countries, such as Egypt, being fat is thought to be a sign of affluence and beauty [33]. Hence, programs that promote awareness and education and emphasize the importance of diet and physical activity are incumbent in developing countries. Studies in the UAE and Saudi Arabia reported non-significant changes in physical activity after the program concluded, despite the improvement in dietary habits and behavior for 6-month durations in these studies [24,37,42], implying the need to identify barriers to physical activity and the necessity of implementing a structured, culturally sensitive physical activity program along with dietary education [21,26]. Cultural sensitivity is defined as the degree to which a target group’s cultural or ethnic features, beliefs, experiences, behavioral patterns, values, norms, and related social, environmental, and historical features are merged into the design, evaluation, and delivery of targeted health promotion programs and materials [76]. This can be achieved through cultural adaptations such as matching materials to group characteristics or targeting the cultural values of the population [76]. Studies in the US also revealed the effectiveness and success of culturally based interventions in promoting a healthy diet and/or physical activity among various ethnicities of participants [77,78,79,80]. Few studies initiated culturally sensitive aspects in their programs, including the use of Arabic language in messages and questionnaires and culturally appropriate examples, such as health beliefs and food habits that represented their respective Arab communities [26,36,37]. Such interventions depicted significant improvements in primary and secondary health outcomes at the end of the program [26,36,37]. However, except one study [26], physical activity outcomes were either not assessed or improved in other studies [36,37]. Barriers to physical activity in Arab countries can occur due to individual factors (e.g., a lack of time or health status), cultural/policy/social factors (e.g., hiring housemaids, traditional roles for women, or not enough social support), and environmental factors (e.g., not enough facilities for exercise or hot weather) [21,22,23,24,25,81,82]. Factors that may promote physical activity are religion (Islamic teachings), the motivation to lose weight, having diseases, exercise benefits, and good social support systems [81,82]. Numerous physical-activity-based interventions aiming to combat the negative impacts of a sedentary lifestyle on health in Arab countries have been published [83,84], indicating the importance of addressing this issue in the region. Hence, there is a necessity to introduce dietary and physical-activity-based programs or policies, considering different genders as well as sociocultural and economic aspects for their success. This also suggests the important role of multidisciplinary teams of dietitians, physicians, lifestyle coaches, and other health professionals in implementing interventions to combat NCDs and promote lifestyle changes linked to physical activity and dietary behaviors to patients through group and individual sessions.

The limitation of this review is that comparisons between studies become challenging due to the differences in the study designs, modes of delivery (online versus face-to-face), and program durations. The durations of the various interventions were less than a year [21,24,26,32,35,37,38,39,40,42], where only two studies performed follow-ups at one year [21,42]. Moreover, only a few studies reported compliance among participants to their respective lifestyle modifications [33,42], while others admitted non-adherence [38,40]. Thus, further studies involving multicomponent interventions with longer durations are required to assess their impacts in managing NCDs and in the long-term maintenance of health outcomes.

## 5. Conclusions

This is the first review to assess diet and physical activity incorporated in their respective interventional programs and how such interventions impact an individual’s lifestyle and work in combating diet-related non-communicable diseases among adults in Arab countries. Personalized, goal-oriented, and longer-duration lifestyle interventions combining diet and physical activity were found to be effective in improving health outcomes. Moreover, considering the mode of delivery for behavioral therapy, a combination of both online and face-to-face sessions was found to be effective and convenient.

Although most interventional studies showed improved health outcomes, some studies did not show any significant differences between the intervention and control groups in terms of physical activity. Hence, it becomes incumbent to identify barriers to physical activity for a culturally acceptable lifestyle intervention program resulting in long-term positive behavioral changes and improvements in health outcomes. The limitations of this review relate to variations in the research design, mode of delivery, and program length. Thus, more studies are needed to assess the effectiveness of multicomponent interventions with longer durations on NCD management.

## Figures and Tables

**Table 1:** Characteristics of lifestyle interventions in Arab countries (*n* = 15).

Author, Year	Country	Target Group and Sample Size	Study Design	Program Duration	Intervention Components	Main Clinical Outcomes
Al Saweer et al., 2017 [32]	Bahrain	Adult female and male employees, Bahraini and non-Bahraini (average age: 46.3 years), N = 97	Prospective cohort study	6 months	Flexible work hours for PA breaks (≥150 mins/week), health club membership discounts, healthier snack options in vending machines, admission to weight reduction programs and nutrition clinics, and periodic meditation sessions for stress management.	Values post- vs. pre-program: Mean BMI (kg/m^2^): 26.72 vs. 28.57 (−1.85 difference); Avg. BP (mmHg): 132/79 vs. 137/82 (−5/−3); Avg. FBS (mmol/L): 4.8 vs. 5.1 (−0.3); Avg. cholesterol (mmol/L): 4.5 vs. 4.58 (−0.08). Increases in fruit and vegetable intake (by 42%) and PA (by 12%) among employees. Decreases in fat intake (by 13%), obesity (by 7%), and vulnerability to stress (by 20%).
Metwally et al., 2019 [33]	Egypt	Type 2 diabetic patients randomly selected (18–70 years, average age: 52.6 years), N = 205	Cohort study	12 months	Total of 48 sessions (one session/week). Interventional lifestyle health education (diet, PA, diabetes, self-monitoring) via multiple integrated techniques, including culturally sensitive and individualized sessions, educational materials, peer education, group therapy, and psychological support.	Positive correlation between HbA1c improvement and healthy nutrition behavior (CC = 0.155, *p* = 0.03), blood glucose monitoring (CC: 0.143, *p* = 0.045), and PA (CC = 0.537, *p* < 0.001). Decreases in barriers (PA, nutrition, and blood glucose monitoring) to diabetes self-management (*p* < 0.001 for all). PA increased after education (*p* < 0.001). Avg. values post- vs. pre-program: HbA1c (%) 8.45 ± 2.46 vs. 11.33 ± 2.02, *p* < 0.001; Weight (kg) 79.23 ± 17.38 vs. 94.28 ± 14.87, *p* < 0.001; BMI (kg/m^2^) 30.35 ± 7.32 vs. 35.13 ± 6.69, *p* < 0.001; WHR 0.97 ± 0.07 and 1.02 ± 0.19, *p* < 0.001.
Alghafri et al., 2018 [34]	Oman	Adult with type 2 diabetes (≥18 years), N = 174	Cluster randomized controlled trial	12 months	IG: Personalized face-to-face consultations with dietitians. PA consultations (≥150 mins moderate or ≥75 mins vigorous or both combined/week, ≥600 MET. min/week). Based on multiple behavioral change techniques. Use of pedometers, accelerometers, and monthly WhatsApp messages. CG: Usual diet and weight management advice, with no PA focus.	No significant changes in HbA1c, BMI, or weight between CG and IG. Decreases in mean SBP (−1.8 mmHg, *p* = 0.04), DBP (−1.6 mmHg, *p* = 0.001), TG (−0.3 mmol/L, *p* = 0.006), sitting time in hours/day (−1.5, *p* < 0.001). Increases in MET.min/week (+447.4, *p* = 0.003) and steps/day (+757, *p* = 0.049) for between-group differences, in favor of IG.
Rashed et al., 2016 [35]	Occupied Palestinian territories	Type 2 diabetes patients (31–70 years), N = 215	Short duration observational study	4 h educational program	Education on DM, disease management (blood glucose monitoring and eye and foot care), BP, smoking cessation, and the importance of PA, dietary management, and weight loss.	Avg. values post- vs. pre-program: BMI (kg/m^2^) 31.23 ± 5.80 vs. 32.1 ± 5.76, *p* = 0.000; Weight (kg) 78.9 ± 1 4.33 kg vs. 80.81 ± 14.95 kg, *p* = 0.000; FBS (mg/dL) 177.7 ± 66.11 vs. 188.65 ± 71.45, *p* = 0.049; HbA1c (%)7.95 ± 1.42 vs. 8.57 ± 1.21, *p* = 0.000; Cholesterol (mg/dL) 169.57 ± 34.23 vs. 183.27 ± 37.74, *p* = 0.000; Triglycerides 183.28 ± 152.4 vs. 209.85 ± 171.04, *p* = 0.025; Knowledge questionnaire score 78.1 ± 13.4 vs. 60.6 ± 20.65; PA not assessed.
Mohamed et al., 2013 [36]	Qatar	Adult patients with Type 2 diabetes (Average age: 55 years for control group; 52 years for intervention group), N = 430	Randomized controlled trial	12 months	IG: Four educational sessions for 3–4 h (on diabetes KAP, PA, nutrition, and counseling). Based on health belief models and theory of empowerment. Idaho plate method (CHO 25%, proteins 25%, vegetables 50%, fruit: one portion, diary: one portion). CG: usual standardized care.	Decreases in mean BMI (−1.70 kg/m^2^, *p* = 0.001), FBS (−0.92 mmol/L, *p* = 0.022), HbA1C (−0.55 mmol/L, *p* = 0.012), and albumin/creatinine ratio (−3.09, *p* < 0.0001). Increases in HDL (0.16 mmol/L, *p* < 0.0001) and KAP (*p* < 0.0001) between groups, favoring IG (in-group comparison). PA not assessed. No significant differences in SBP, DBP, TG, LDL, or total cholesterol between groups.
Alfawaz et al., 2019 [39]	Saudi Arabia	Adult Saudis with prediabetes (20–60 years), N = 160	Multi-center 6-month interventional study	6 months	Guidance group: well-structured and monitored nutrition and lifestyle counseling about diabetes, PA, nutrition, and weight management. GA group: a one-time GA about lifestyle modification.	In group comparisons (guidance vs. GA): FBS (mmol/L) 5.70 ± 1.0 vs. 5.87 ± 1.1, *p* = 0.005; HbA1c (%) 5.35 ± 1.0 vs. 5.41 ± 1.1, *p* = 0.005; Exercise (vigorous PA/week) 1.40 vs. 0.6, *p* < 0.017; BMI (kg/m^2^): No statistical change. Recommended intakes of total carbohydrate (46.9% compliance post-program vs. 20.3% at baseline, *p* = 0.003); dietary fiber (21.9% vs. 3.1%, *p* = 0.002); and micronutrients (vitamin B2 (*p* < 0.01), B3 (*p* < 0.01), B6 (*p* = 0.01), B12 (*p* = 0.04), C (*p* = 0.02), Mg (*p* = 0.02), Fe (*p* = 0.03), and Cu (*p* < 0.01)) improved in guidance group but not GA.
Alghamdi, 2017 [38]	Saudi Arabia	Arab and Saudi adult obese patients (females and males, age ≥ 20 years), N = 140	Randomized clinical trial	3 months	ILI: Eight clinical visits to attain significant weight loss (≥5%). Individualized lifestyle intervention based on USPSTF guidelines. Avg. CHO intake (20–25 g/day) based on Atkins diet. PA target: ≥150 min/week. AC: One education session on diet and PA, with no behavioral support.	Mean weight loss of ≥5% in ILI only (*p* < 0.001). Inter-group comparison showed decreases in mean weight (−2.77 kg, *p* = 0.002), BMI (−1.09 kg/m^2^, *p* = 0.002), WC (−2.13 cm, *p* = 0.01), hip circ. (−2.06 cm, *p* = 0.03), and DBP (−2.44 mmHg, *p* = 0.01) favoring ILI but not SBP (*p* = 0.06). Diet and PA were not measured as health outcomes.
Al-Hamdan et al., 2019 [24]	Saudi Arabia	Prediabetic obese or overweight females (18–55 years), N = 123	Interventional study	7 months	IG: one-on-one intensive lifestyle modification sessions on weight decrease (5% from baseline), PA (4 h/week), dietary counseling on decreasing fat intake (30% and 10% of total energy for total and saturated fat) and increasing fiber intake (15 g/1000 kcal). CG: standard guidance.	Significant decreases in SBP (121.9 ± 9.3 mmHg in IG vs. 127.4 ± 13.6 mmHg in CG, *p* = 0.01), total cholesterol (4.7 ± 1.0 mmol/L vs. 4.5 ± 0.8 mmol/L, *p* = 0.04), energy and macronutrient intake (*p* < 0.001), HbA1c levels (5.8 ± 0.3% vs. 6.3 ± 0.4%, *p* < 0.001), and WHR (0.83 ± 0.09 vs. 0.86 ± 0.08, *p* = 0.04) and an HDL increase (1.8 ± 0.5 mmol/L vs. 1.1 ± 0.3 mmol/l, *p* < 0.001) (between-group comparisons). No significant differences in BMI, PA, weight, DBP, TG, or LDL between groups.
Khouja et al., 2020 [40]	Saudi Arabia	Women having a moderate to high risk of CVD (age: ≥30 years, mean age: 42 ± 8 years), N = 59	Randomized controlled trial	3 months	IG: A 2 h visit, one session/week for 4 weeks on diet counselling, exercise training (≥30 mins/day), and health education, individually and in groups. Monthly monitoring via phone following national guidelines for cardiometabolic risk factor management. CG: standard care and one health education session.	Significant mean differences in SBP (−9.2 mmHg, *p* = 0.01), FRS (−13.6, *p* < 0.01), and blood glucose level (−45 mg/dL, *p* = 0.03) between groups, favoring IG, but no significant difference in WC, blood lipid levels, or BMI. Diet and PA were not measured as health outcomes.
Sani et al., 2018 [37]	Saudi Arabia	Newly detected diabetes mellitus patients (≥18 years), N = 200	Quasi-experimental two-group pre- and post-evaluation study design	6 months	IG: Peer groups with monthly meetings, periodic messages in Arabic (2X/week), and discussions. Problem-based learning techniques. Practical sessions on purchasing options, cooking techniques, PA, and self-management of DM. CG: usual standard care.	Differences in mean HbA1C (−16.87%, *p* = 0.000), BMI (− 5.49%, *p* = 0.000), SBP (− 6.07%, *p* = 0.001), and total cholesterol values (−9.97%, *p* = 0.016) between IG and CG. No significant improvement in PA, FBS, triglycerides, LDL, DBP, or HDL (*p* > 0.05). Significant improvement in diabetes knowledge in IG in comparison to CG (*p* = 0.001).
Wani et al., 2020 [28]	Saudi Arabia	Overweight/obese Saudi adults with prediabetes (≥20 years), N = 300	Randomized controlled study	12 months	IG: Individualized self-monitored lifestyle modification program on food choices, diet (total dietary fat < 30% of energy, fiber intake 15 g/1000 kcal), PA (≥5000 steps/day), and weight loss by dietitian and physician. Follow-up via message/email/call. GA: standardized care.	Weight (kg) 78.01 ± 15.8 in IG vs. 83.27 ± 13.7 in GA (≥5% weight loss), *p* < 0.01; BMI (kg/m^2^) 30.57 ± 6.3 in IG vs. 33.39 ± 5.9 in GA, *p* < 0.01; FBS (mmol/L) 5.59 ± 0.8 in IG vs. 5.92 ± 0.8 in GA, *p* < 0.01. No significant decrease in lipid levels.Diet and PA were not measured as health outcomes.
Bhiri et al., 2015 [41]	Tunisia	Adult males and female employees (Avg age: 33.86 ± 8.10 years in intervention group, and 38.90 ± 8.77 in control group), N = 1775	Quasi-experimental study	4 years	IG: Health education programs, including workshops, films, and open sensitization days. Healthy diet sessions (five fruit and vegetable servings/day), PA sessions (≥150 mins moderate or ≥75 mins vigorous or both PA/ week), and smoking cessation consultations. CG: no intervention.	Behavior assessment: Recommended fruit and vegetable intake: increased from 47.5% to 52.1% of participants in IG only (*p* = 0.04). Recommended PA: increased from 28.3% to 37.9% in IG (*p* < 0.001) and from 31.2% to 42.9% in CG (*p* < 0.001).
Sadiya et al., 2016 [21]	United Arab Emirates	Adult men and women obese with/without Type 2 Diabetes (mean age: 42 years), N = 45	Intervention program	3-month intervention (follow-up after 1 year of maintenance phase)	Eight sessions (three individual and five group sessions) combining behavioral therapy, PA, and diet modification to attain up to 5% weight loss. Self-management skills and personalized goals supervised by registered dietitian. Diet plans (1200–1500 kcal/day) as per ADA guidelines, including intake of cereals (5–7 servings/day) and vegetables (3–4 servings/day). Exercise sessions (45 mins, 2X/week), including moderate PA (150–250 mins/week) or 7000–10,000 steps/day.	Avg. values post- vs. pre-program: BMI (kg/m^2^) 38.4 ± 7.4 vs. 40.4 ± 7.4, *p* < 0.01; Weight (kg) 93.4 ± 19.4 vs. 98.2 ± 19.4, (5% loss), *p* < 0.01; WC (cm)106 ± 14 vs. 110 ± 14, *p* < 0.01; FBS (mmol/L) 6.8 ± 0.8 vs. 8.2 ± 2.0, *p* < 0.05; HbA1c (%) 6.6 ± 0.7 vs. 7.1 ± 1.0, *p* < 0.05. One-year follow-up: sustained FBS (6.6 ± 1.4 mmol/L, *p* < 0.05), HbA1c (6.3 ± 0.7%, *p* < 0.05), and weight loss (−4.0% from baseline, *p* < 0.001). Nutritional knowledge increased (*p* < 0.01). PA was not assessed.
Abdi et al., 2015 [42]	United Arab Emirates	Overweight or Obese Adult patients with Type 2 Diabetes (18–60 years), N = 35	Translational randomized controlled trial with two parallel arms	6-month lifestyle program (follow-up after 1 year of maintenance phase)	IG: Eight sessions (four individual sessions and four telephone calls by clinical dietitians) of cognitive behavioral theory (CBT)-based nutritional counseling with nutrition (1200–1800 kcal) and PA targets (≥30 mins, 5X/week). CG: received standard care. One-year follow-up for all.	Decrease in HbA1c (by −1.17 ± 2.11 in IG, *p* = 0.000) at 6 months, maintained at 1 year (−1.12 ± 1.46, *p* < 0.05) in IG only. CHO intake reduced from total CHO and cereals by 20.94 ± 56.73 g/day and 32.92 ± 54.34 g/day (*p* < 0.05) in IG only. No significant changes in lipid profiles, DBP, SBP, mean weight, PA, or BMI in either group (*p* > 0.05) after intervention.
Ali et al., 2021 [26]	United Arab Emirates	Female overweight or obese students from two universities (18–35 years), N = 246	Non-randomized two-arm feasibility study	4 months	R-Enhanced: A social cognitive theory-based program, using a website and mobile apps. Self-monitoring and tracking diet and PA with apps. Support and coaching from dietitians to meet goals. R-Basic: Access to a static website with educational material on diet and PA.	No significant difference in weight loss or BMI between groups. Median WC decrease (from 91 cm to 86 cm (baseline to endline), *p* = 0.003), higher nutrient-source knowledge (*p* < 0.001), increased days with moderate PA (*p* = 0.013), mins walked (*p* < 0.001), and diet–disease relationships (*p* = 0.006) in R-Enhanced group only. Higher scores in social support reduced fat intake (*p* = 0.006) and increased PA (*p* = 0.001).

Abbreviations: AC: Education-Only Active Comparator; BMI: Body Mass Index; BP: Blood Pressure; CC: Correlation Coefficient; CG: Control Group; CHO: Carbohydrates; Cu: Copper; CVD: Cardiovascular Disease; DBP: Diastolic Blood Pressure; FBS: Fasting Blood Sugar; Fe: Iron; FRS: Framingham Risk Score; GA: General Advice; HbA1c: Glycosylated Hemoglobin; HDL: High-Density Lipoprotein; IG: Intervention Group; ILI: Intensive Lifestyle Intervention, KAP: Knowledge, Attitude, and Practice; LDL: Low-Density Lipoprotein; MET: Metabolic Equivalent; Mg: Magnesium; PA: Physical Activity; SBP: Systolic Blood Pressure; TG: Triglycerides; USPSTF: US Preventive Services Task Force; WC: Waist Circumference; WHR: Waist/Hip Ratio.

## Data Availability

Data sharing is not applicable.
